# Multiple Consecutive Cervicovaginal Cytology Specimens Confirm Persistent Colonization by *Cokeromyces recurvatus*: Case Report and Literature Review

**DOI:** 10.1155/2022/2151926

**Published:** 2022-05-13

**Authors:** Keng Lor, Christopher P. Hartley, Bobbi S. Pritt, Anna M. Kemp, Amy A. Swanson, Charles D. Sturgis

**Affiliations:** ^1^Department of Pathology and Laboratory Medicine, Mayo Clinic, Rochester, MN, USA; ^2^Department of Pathology and Laboratory Medicine, Mayo Clinic, La Crosse, WI, USA

## Abstract

The published literature on cervicovaginal cytology includes fewer than ten reported cases of *Cokeromyces recurvatus* identified in Pap test samples. We report a unique case of an asymptomatic 27-year-old female with persistent gynecologic tract colonization by *C. recurvatus* in which distinctive fungal microorganisms were identified in three samples collected over three consecutive years.

## 1. Introduction

The dimorphic fungus *Cokeromyces recurvatus* is a member of the Mucoraceae family from the order Mucorales. While certain organisms in the Mucoraceae group may cause serious illnesses, such as rhinocerebral and pulmonary mucormycosis, with mortality rates exceeding 90 percent, other organisms in this family such as *C. recurvatus* are most often encountered as incidental findings in otherwise asymptomatic individuals [1]. Each year in the United States, approximately 50 million cervicovaginal cytology cancer screening tests (Pap tests) are performed. A review of the literature confirms only seven reported cases of *C. recurvatus* in cervicovaginal cytology specimens; each of these being described in the last 25 years [2–7]. In all of the previously reported cases of *C. recurvatus* from cervicovaginal cytology specimens, no patients have been shown to have invasive fungal disease in the gynecologic tract; however, one pregnant patient with clinical cervicitis attributed to coinfection with *C. recurvatus* and *Candida albicans* was successfully treated with antifungal therapy [5]. Morphologic identification of *C. recurvatus* and separation of this organism from the important morphologic differential diagnosis of *Paracoccidioides brasiliensis* may be important for patient management. Herein, we describe a unique case of persistent, asymptomatic *C. recurvatus* cervicovaginal colonization in a healthy young woman with involvement of 3 consecutive annual liquid-based Pap tests.

## 2. Case Presentation

**Figure 1:**
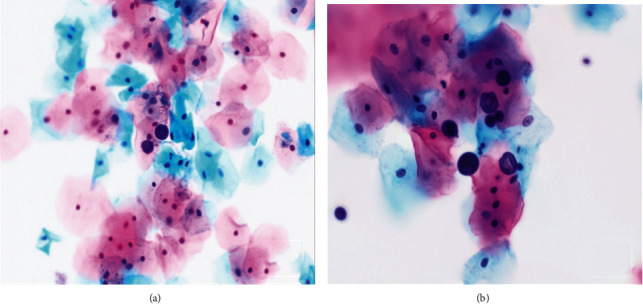
(a) Two large (10 to 30 *μ*m) fungal organisms at intermediate magnification. (b) Multiple large (10 to 30 *μ*m) fungal organisms, some with peripheral daughter buds (2 to 4 *μ*m) at high magnification. Background intermediate and superficial squamous epithelial cells provide valuable size references (cervicovaginal cytology, ThinPrep, Papanicolaou stain 400x (a), 600x (b)).

**Figure 2:**
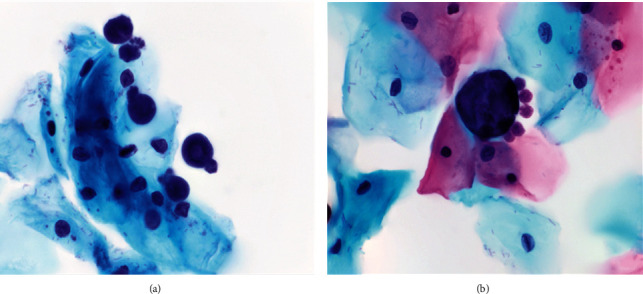
(a) Multiple globular and large (10 to 30 *μ*m) fungal organisms. (b) Single large rounded fungal element with numerous asymmetrically distributed peripheral daughter buds (2 to 4 *μ*m), morphology confirmed as *Cokeromyces recurvatus*. In close proximity are superficial and intermediate squamous epithelial cells (cervicovaginal cytology, ThinPrep, Papanicolaou stain 1000x (a, b)).

**Figure 3:**
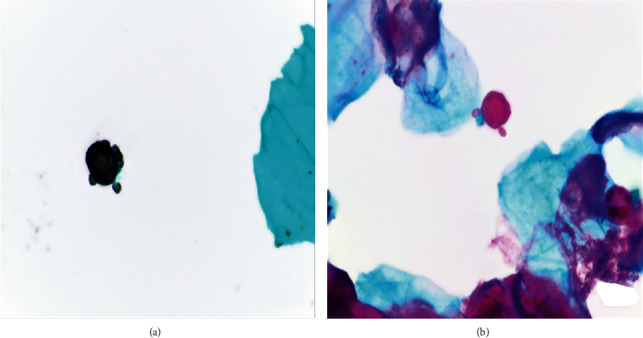
(a) Single large *C. recurvatus* fungal microorganism with multiple peripheral daughter buds. (b) Another organism with 2 peripheral daughter buds with background squamous epithelial cells (cervicovaginal cytology, ThinPrep, Gomori methenamine silver stain 1000x (a), periodic acid–Schiff stain 1000x (b)).

The patient was a 27-year-old female with no known history of cervical intraepithelial neoplasia or sexually transmitted diseases. She presented for annual cancer screening, and an exfoliative cervicovaginal cytology collection was performed. The sample was submitted in a liquid-based vial. A single ThinPrep Pap test slide was created using manufacturer's guidelines (Hologic, Ontario, Canada). The reviewing cytotechnologist and pathologist discovered large globoid fungal elements of uncertain type. They performed Gomori methenamine silver (GMS) and periodic acid–Schiff (PAS) stains (Figures 1–3). They also retrieved 2 previous Pap test slides from this patient from their archival files. In retrospect, these organisms had been present for 3 consecutive years. Expert consultation was sought, and all slides with accompanying reports were sent to the Mayo Clinic cytopathology consultation service. The slides were reviewed at Mayo Clinic, Rochester, with assessments performed by cytopathologists and a medical microbiologist. The diagnosis of *Cokeromyces recurvatus* was confirmed based upon light microscopic evaluations.

## 3. Discussion

Three consecutive annual cervicovaginal screening slides were received from an out of state laboratory for expert extradepartmental consultation. All 3 slides were liquid-based cytology ThinPrep studies. These specimens had been collected in consecutive calendar years: 2019/2020/2021. Routine mechanized ThinPrep processing had been employed. The slides were received for consultation and were reviewed by a cytotechnologist and a cytopathologist with consultations from additional cytopathologists and a medical microbiologist.

All 3 Pap slides were of adequate cellularity and included appropriate endocervical components. All 3 slides were interpreted as negative for intraepithelial lesion or malignancy (NILM). Large and globular organisms (ranging from 15 to 35 *μ*m) were seen in all 3 samples (Figures 1 and 2). Some but not all of these organisms showed asymmetrically distributed peripheral daughter buds. GMS and PAS studies confirmed the organism to be fungal with appropriate cell wall staining characteristics (Figure 3). The light microscopic Papanicolaou stain and special stain results were diagnostic of the yeast-like stage of *Cokeromyces recurvatus*.

In light microscopic slides from human specimens, *C. recurvatus* may be mistaken for the yeast stage of another dimorphic fungus, *Paracoccidioides brasiliensis*, due its size and resemblance, with peripheral budding. *P. brasiliensis* yeasts have been classically described as having a morphologic “mariner-wheel pattern” in cytologic and histologic preparations. *P. brasiliensis* is a pathogenic fungus that can induce significant clinical diseases, and it is primarily reported in Central and South America with most cases in Brazil, Columbia, and Venezuela [7]. Though *C. recurvatus* may display similar morphology to that of *P. brasiliensis* in cytology slides, the peripheral buds of *P. brasiliensis* are better described as symmetrically distributed, while the budding of *C. recurvatus* is classically asymmetrical and patchy. The patient in this case resided in the U.S. state of Georgia, living outside of the expected geographic distribution for most cases of disease associated with *P. brasiliensis*. She had no known travel history to regions of *P*. *brasiliensis* endemicity. Although molecular and mycologic confirmations of *C*. *recurvatus* are possible, only 3 stained slides were available for use, and the ThinPrep vials were not accessible for ancillary confirmative studies. Molecular confirmation of esoteric organisms is known to be challenging in referral expert extradepartmental consultation services when only slides are received.

Cervicovaginal cytology specimens harboring the yeast-like form of *C. recurvatus* organisms have been reported in the literature in 7 cases, and 4 of those cases had cultures confirming the fungal organism [2, 5, 6, 7]. When identified outside of the gynecologic tract, *C. recurvatus* has been known to cause hemorrhagic cystitis in a 72-year-old, diverticulitis in a 9-year-old, and fatal pneumonia in an immunocompromised 27-year-old [8–10]. While nongynecologic infections with *C. recurvatus* may be associated with significant clinical disease, patients with this fungus reported in cervicovaginal cytology specimens are most often asymptomatic without symptoms or signs of disease. A single 31-year-old pregnant patient with clinical vaginitis and vulvar lichen simplex chronicus was previously reported with fungal cultures revealing both *Candida albicans* and *C. recurvatus*. This patient was successfully treated with antifungal therapy, intravaginal terconazole [5]. One previous patient, a 37-year-old asymptomatic female, was described to have *C. recurvatus* organisms present in cervicovaginal cytology specimens prepared 1 year apart, highlighting that *C. recurvatus* may be capable of long-term colonization of the female genital tract [2].

In conclusion, we report a rare case of persistent *C. recurvatus* colonization in the gynecologic tract of a healthy and otherwise asymptomatic young adult woman. Diagnostic organisms were present in multiple samples collected over three consecutive years. While the clinical significance and origins of these organisms in this patient are uncertain, it is important to accurately diagnose fungal organisms such as *C. recurvatus* to separate them from other morphologically similar and potentially pathogenic fungi, such as *P. brasiliensis*. It may be of value to not only identify *C. recurvatus* in cervicovaginal cytology reports but also to mention that these organisms may be nonpathogenic colonizers, as it is important to treat the patient and not a laboratory finding.

## Data Availability

All data used in this manuscript are included in the case report.
